# Massive hemoptysis controlled with transection of a pulmonary vein and bronchus-a case report

**DOI:** 10.1186/1749-8090-8-209

**Published:** 2013-11-11

**Authors:** Hui-Ju Ho, Ching-Yuan Cheng, Bing-Yen Wang

**Affiliations:** 1 Department of Surgery, Division of Thoracic Surgery, Changhua Christian Hospital, and Institute of Medicine, Taichung, Taiwan; 2 Chung Shan Medical University, Taichung, Taiwan; 3 The Department of Medicine of The National Defense Medical Center, Taipei, Taiwan; 4 School of Medicine, National Yang-Ming University, Taipei, Taiwan

**Keywords:** Massive hemoptysis, Bronchiectasis, Partial lung resection

## Abstract

Massive hemoptysis caused by bronchiectasis in which bronchial artery embolization does not control the bleeding is not rare. Traditional surgical intervention is anatomical lung resection. We present a case of a patient with bronchiectasis and massive hemoptysis in which the bleeding was controlled with transection of a pulmonary vein and bronchus with preservation of the pulmonary artery.

## Background

Massive hemoptysis is a life threatening condition, and the treatments include bronchoscopic intervention, bronchial artery embolization, and surgical intervention. Some case reports of controlling hemoptysis by either division of the bronchus or ligation of the pulmonary artery for the control of bleeding have been published. We report a case with massive hemoptysis which was treated successfully by only transecting the pulmonary vein and bronchus.

## Case presentation

**Figure 1:**
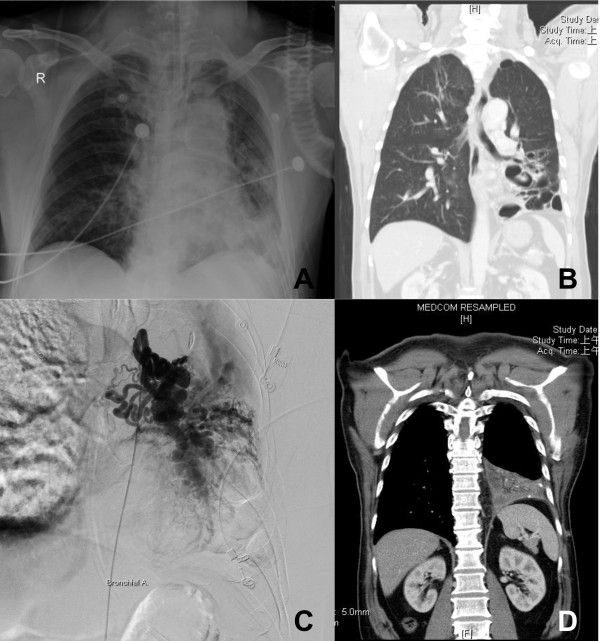
**Some related images before and after the operation. A)** Chest plain radiograph showed opacity in the left lower lobe with bronchial dilatation. **B)** Chest computed tomography (CT) revealed cystic dilatation in lingula and left lower lobe with numerous small vessels in the left hilar region. **C)** Angiography from the left bronchial artery revealed dilated and tortuous vessels. **D)** Chest CT 3 months postoperatively revealed consolidation of the left lower lobe and the absence of numerous prolifeating vessel.

A 57-year-old Asia male with a history of bronchiectasis presented with sudden onset of massive hemoptysis. The patient was sent to our emergent department and emergent endotracheal intubation was performed for airway protection. Chest plain radiography showed increasing opacity in the left lower lobe with bronchial dilatation (Figure 1A). Chest computed tomography (CT) demonstrated bilateral bronchitis with cystic dilatation in the lingula and left lower lobe with numerous small vessels in the left hilar region (Figure 1B). Bronchoscopy revealed a large amount of fresh blood over the lower trachea, and the examination could not be completed. Pulmonary angiography demonstrated a plexus of proliferating vessels around left bronchus artery (Figure 1C).

After adequate resuscitation with blood products, embolization of the left brachial artery was performed. However, some proximal collateral vessels which could not be embolized remained. After the procedure, persistent hemoptysis was noted, and therefore surgical intervention was taken.

After induction of general anesthesia, the patient was intubated with a double-lumen endotracheal tube and placed in the right decubitus position. Two trocars were inserted at 6th and 8th intercostal spaces, respectively, and a 15-cm thoracic incision in the 5th intercostal space was made and a rib retractor was placed. Severe adhesions in the pleural space were found, and all were detached. The major fissure and pulmonary artery were difficult to identify because of severe consolidation of the left lung. The border between the left upper lobe and left lower lobe was unclear. Enlargement and marked tortuosity of the bronchial arteries were also found, and these vessels were ligated with sutures. Pneumonectomy was considered, however, it was not performed in consideration of the high potential mortality. The inferior pulmonary vein was transected to allow identification of the left lower lobe bronchus. The bronchus of the left lower lobe was then transected to control the hemoptysis, and the pulmonary artery was preserved. Two 32F straight chest tubes were placed after checking for bleeding and air-leakage.

No hemoptysis was noted postoperatively, and the patient’s fever subsided by postoperative day 10. The chest tubes were removed on postoperative day 18, and he was discharged on postoperative day 24. The postoperative course was uneventful.

**Figure 2:**
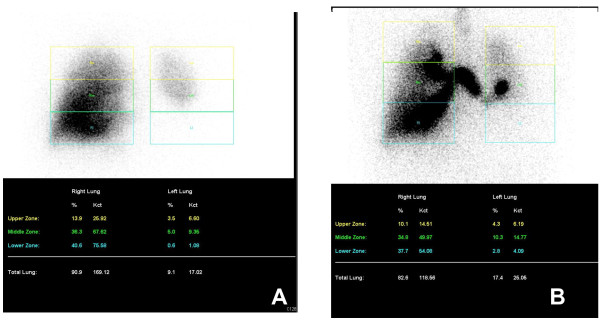
Ventilation-perfusion scan (v/q scan) 3 months postoperatively which demonstrated (A) perfusion and (B) ventilation deficits.

Follow-up chest CT 3 months postoperatively demonstrated cystic dilatation in lingula with numerous small vessels in the left hilar region and some fluid accumulation in the pleural cavity (Figure 1D). Lung perfusion scan and aerosol ventilation study revealed deficits in perfusion and ventilation (Figure 2). The transacted bronchus contributed to the ventilation deficit. A possible explanation of the perfusion deficit may be that the resected pulmonary vein blocks blood flow, whereas the intact pulmonary artery maintains blood inflow thus causing some embolus formation resulting in a lack of perfusion. The patient exhibited no signs or symptoms of infection, and hemoptysis had not recurred.

## Discussion

Hemoptysis is a common symptom in bronchiectasis patients. However, other causes of hemoptysis include tuberculosis, mycetomas, necrotizing pneumonia, and bronchogenic carcinomas [1,2]. Treatments for massive hemoptysis include cold saline lavage, epinephrine, endobronchial stent tamponade, bronchial artery embolization, and invasive surgical intervention. The standard surgical procedure for the treatment of hemoptysis is anatomic lung resection (pneumonectomy, lobectomy, segmentectomy). Nonanatomic (wedge) resection should be avoided due to greater risk of failure to control the bleeding [3,4].

In our initial purpose, anatomic resection of lung (left lower lobe lobectomy) was planned to do. Due to severe bronchiecstasis and inflammation, there’s much extremely tortuous and engorged collateral arteries around. We therefore decided to resect the pulmonary vein first and then the bronchus and pulmonary artery sequentially. But the severe adhesion fissure and tortuous vessels made the dissection of pulmonary artery unachievable after the bronchus resected. What’s more, the border of left upper and lower lobe also got severe inflammation and adhesion. Pneumonectomy seems the only way to get anatomic resection of lung in this patient. However, high risk and postoperative mortality was concerned and we decided to leave the pulmonary artery alone and perform a large wedge resection with both pulmonary vein and bronchus resected.

**Figure 3:**
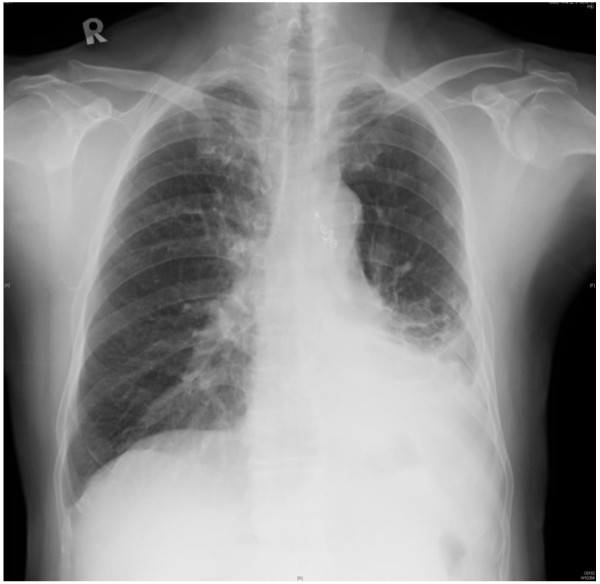
At 10 months postoperatively chest plain film sowed no progression in left lower lobe opacity.

Both infection and bleeding are major concerns in this patient postoperatively. As for infection, we elongated the use of antibiotics to 4 weeks. And for bleeding, there’s no hemoptysis after the procedure or at further outpatient clinics follow-up. This patient can tolerate daily activity and some light works after discharge. The chest plain film also revealed no obvious progression ten months after the operation (Figure 3).

On searching related articles, we found no similar case reports. The only related procedure we found was resection of the pulmonary artery and bronchus whereas leave the pulmonary vein [5]. In that procedure, the isolated lung continues to receive a blood supply from the vessels between the lung surface and the chest wall, which keeps the lung parenchyma viable. Moreover, the intact pulmonary veins drain the blood, and therefore no necrosis of the lung parenchyma occurs [5]. In our case, the pulmonary artery was kept intact, and the pulmonary vein was resected. The bronchus of the left lower lobe was transected to control the hemoptysis. We considered that pneumonectomy for this patient was technical feasible to control the hemoptysis, but the associated risk of mortality was high. We wanted to preserve the lung function of left upper lobe, and therefore we resected the inferior pulmonary vein and bronchus and left the pulmonary artery. In follow-up imaging studies asymptomatic local lung consolidation with deficits in ventilation and perfusion were noted.

## Conclusions

A combined ligation of the pulmonary vein and the bronchus to produce physiological lung exclusion for the control of hemoptysis hasn’t been reported. This case illustrates that this is a therapeutic option for controlling massive hemoptysis.

## Consent

Written informed consent was obtained from the patient for publication of this case report and any accompanying images. A copy of the written consent is available for review by the Editor-in-Chief of this journal.

## Competing interests

All the authors declare that they have no competing interests.

## Authors’ contributions

In the following we specify the individual contributions of authors to the manuscript. All surgical procedures were performed by BYW, HJH and CYC. BYW and HJH managed the perioperative period of the patient. HJH prepared the manuscript and BYW made final approval. All authors read and approved the final manuscript.
